# Changes in the Peripheral Blood Gene Expression Profile Induced by 3 Months of Valproate Treatment in Patients with Newly Diagnosed Epilepsy

**DOI:** 10.3389/fneur.2015.00188

**Published:** 2015-08-31

**Authors:** Aleksei Rakitin, Sulev Kõks, Ene Reimann, Ele Prans, Sulev Haldre

**Affiliations:** ^1^Department of Neurology and Neurosurgery, University of Tartu, Tartu, Estonia; ^2^Neurology Clinic, Tartu University Hospital, Tartu, Estonia; ^3^Department of Pathophysiology, University of Tartu, Tartu, Estonia; ^4^Centre of Translational Medicine, University of Tartu, Tartu, Estonia

**Keywords:** valproic acid, valproate, gene expression, epilepsy, teratogenicity

## Abstract

Valproic acid (VPA) is a widely used antiepileptic drug with a broad range of effects and broad clinical efficacy. As a well-known histone deacetylase (HDAC) inhibitor, VPA regulates epigenetic programming by altering the expression of many genes. The aim of study was to analyze differences in gene expression profiles before and after the start of VPA treatment in patients with newly diagnosed epilepsy. RNA sequencing was used to compare whole-genome gene expression patterns of peripheral blood from nine patients with epilepsy before and 3 months after the start of treatment with VPA. Of the 23,099 analyzed genes, only 11 showed statistically significant differential expression with false discovery rate-adjusted *p*-values below 0.1. Functional annotation and network analyses showed activation of only one genetic network (enrichment score = 30), which included genes for cardiovascular system development and function, cell morphology, and hematological system development and function. The finding of such a small number of differently expressed genes between before and after the start of treatment suggests a lack of HDAC inhibition in these patients, which could be explained by the relatively low doses of VPA that were used. In conclusion, VPA at standard therapeutic dosages modulates the expression of a small number of genes. Therefore, to minimize the potential side effects of HDAC inhibition, it is recommended that the lowest effective dose of VPA be used for treating epilepsy.

## Introduction

Valproate [also known as valproic acid (VPA)] is one of the most frequently used antiepileptic drugs worldwide (1) and is widely applied for other conditions, such as migraine prophylaxis and bipolar disorder (2). Usually, VPA is used for treating chronic conditions requiring long-term treatment, which emphasizes the importance of the long-term safety of the drug. VPA has a broad range of effects and broad clinical efficacy, leading to its frequent designation as a “dirty drug” (3). VPA acts directly at the level of gene transcription by inhibiting histone deacetylation (HDAC) and increasing access to transcription sites (4). Recent data have shown that HDAC inhibitors and VPA regulate innate and adaptive immune pathways, with possible anti-inflammatory effects (5). The use of VPA during pregnancy has been associated with an approximately two to threefold increase in the rate of major anomalies, such as spina bifida, as well as cardiac, skeletal, craniofacial, and limb defects. VPA-induced inhibition of HDAC can result in cell-cycle disruption, growth arrest, and apoptosis, which may explain the teratogenic action of the drug (6).

In *in vitro* and *in vivo* preclinical studies, VPA showed strong antitumor effects in a wide variety of cancers by modulating multiple pathways, including cell-cycle arrest, angiogenesis, apoptosis, and differentiation (4). VPA inhibits the proliferation and induces the differentiation of malignancies, such as leukemia, lymphoma, teratocarcinoma, and medulloblastoma. Clinically, VPA has been used to treat leukemia and some solid tumors (7). About half of patients treated with VPA report weight gain, associated with important metabolic and endocrine abnormalities (8). Although the molecular mechanisms underlying VPA-induced weight gain are unknown, VPA was shown to suppress adiponectin gene expression in adipocytes (9). Accumulating evidence suggests that endoplasmic reticulum stress plays a role in the pathogenesis of type 2 diabetes, obesity, and insulin resistance (10). VPA modulates this stress response through the increased expression of members of the endoplasmic reticulum stress protein family, such as WFS1 (11), GRP94, and calreticulin (12).

As is evident, VPA is able to modulate the expressions of genes in different molecular pathways. This study was designed to evaluate the effect of 3 months of VPA treatment in patients who were recently diagnosed with epilepsy, by analyzing the differences in gene expression profiles before and after the start of treatment. Gene expression profiling in these patients could provide clues for understanding the molecular mechanisms of various side effects of VPA, such as metabolic changes, teratogenicity, and others.

## Materials and Methods

### Subjects

Nine otherwise healthy subjects (age range: 18–38 years) with newly diagnosed epilepsy and for whom VPA treatment was indicated were considered for enrollment in a case-crossover study on the effect of VPA on peripheral blood RNA expression. Patients were excluded if there was history or evidence of non-compliance with medical regimens; treatment with any medication other than VPA, including hormonal contraception; evidence of a cardiovascular, hepatic, renal, oncologic, or progressive neurological disease that could have an impact on blood RNA expression; evidence of progressive lesions on computed tomography or magnetic resonance imaging; or if the patient was pregnant.

All patients were clinically examined and interviewed by the first author. The main subject characteristics, including epileptic syndrome and VPA dose, are summarized in Table 1. Before and 3 months after the start of VPA treatment, the weight and height were measured, and the body mass index (BMI) was calculated as the weight (in kilogram) divided by the square of height (in meter). Seizures and epileptic syndromes were classified by using the criteria proposed by the ILAE (13). No patients had epileptic seizures during the evaluation period.

**Table 1:** **Clinical and demographic characteristics of patients**.

Sex	Age (years)	Epilepsy etiology	Daily VPA dose (mg/d)	Daily VPA dose (mg/kg)	BMI before treatment (kg/m^2^)	BMI after treatment (kg/m^2^)
F	38	Gen	600	12.5	21.3	21.8
F	38	Str/met	900	15	21.8	21.8
M	21	Gen	900	8.3	34.5	34.5
M	21	Gen	600	6.9	24.1	25.5
M	18	Gen	600	9.7	20.0	23.6
F	21	Gen	600	12.8	17.7	18.8
F	33	Gen	600	11.8	20.2	21.0
F	18	Gen	600	9.8	19.5	19.8
F	32	Str/met	1200	15.8	24.8	24.5

*Gen, genetic; Str/met, structural/metabolic; VPA, valproate; BMI, body mass index*.

### Sample collection and RNA preparation

Blood samples were collected during two visits, before and around 3 months after the start of treatment with VPA. The daily VPA dose was 600–1200 mg (mean dose: 11.4 ± 2.8 mg/kg). The study protocol and consent forms were approved by the Ethics Committee on Human Research at Tartu University. Blood samples were collected into Tempus tubes (Applied Biosystems, Foster City, USA). Blood was frozen and stored until further processing. RNA was extracted from whole blood with an RNA extraction kit, in accordance with the manufacturer’s protocol (Applied Biosystems).

### Statistical analysis

Differential gene expression was analyzed by using the EdgeR package with non-normalized raw counts after the quality control of samples. EdgeR is a very flexible tool for RNA sequencing data analysis, which uses model-based scale normalization, dispersion estimates, negative binomial model fitting, and testing procedures to determine the differential expression of genes (14, 15). As our sample contains paired samples (pre- and post-VPA), a paired testing approach was used. General linear modeling was applied, with subjects being added to the contrast matrix. The general linear modeling likelihood ratio test was applied to compare pre- and post-VPA results. Sequencing of the fragment library resulted in an average of 15 million reads per sample. False discovery rate adjustment was used for multiple testing corrections (16). The threshold for statistical significance was an adjusted false discovery rate of 0.1.

### Functional analysis of differentially expressed genes

Functional network analysis is used to identify the biological functions that are most significantly related to the molecules in a network. To define the functional networks of differentially expressed genes, the data were analyzed by using the Ingenuity Pathway Analysis (Ingenuity Systems, www.ingenuity.com), which calculates a significance (network) score for each network. This score indicates the likelihood that the assembly of a set of focus genes in a network could be explained by random chance alone (e.g., a score of 2 indicates that there is a 1 in 100 chance that the focus genes are together in a network due to random chance). A data set containing the Affymetrix probe-set identifiers and their corresponding fold change (log_2_) values was uploaded into the Ingenuity Pathway Analysis software. Each gene identifier was mapped to its corresponding gene object in the Ingenuity Pathways Knowledge Base to identify molecules whose expression was significantly differentially regulated. These focus genes (or Network Eligible molecules) were overlaid onto a global molecular network developed from information contained in the Ingenuity Pathways Knowledge Base. Networks of these focus genes were then algorithmically generated on the basis of their connectivity.

A network is a graphical representation of the molecular relationships between genes or gene products (represented as nodes). The biological relationship between two nodes is represented as an edge (line). All edges are supported by at least one reference from the literature, or from canonical information stored in the Ingenuity Pathways Knowledge Base.

## Results

Comparison of blood RNA samples isolated from patients with epilepsy before and 3 months after the start of VPA treatment revealed distinct gene expression profiles. Altogether, 11 of the 23,099 analyzed genes showed statistically significant differential expression with multiple testing-adjusted false discovery rate values less than 0.1 (Table 2). Relative differences in the expression signal (fold change or log fold change) between two time points were generally low. After initiation of VPA treatment, only two genes were up-regulated more than 1.1-fold, and no genes were down-regulated more than 1.1-fold, compared to the status before VPA treatment.

**Table 2:** **Most significantly up- or down-regulated genes after exposure to valproate**.

Gene symbol	Log(FC)	*p*	FDR	Gene name
TPT1	−0.14	2.00 × 10^−8^	0.00046105	Tumor protein, translationally controlled 1
HBG2	0.86	3.16 × 10^−7^	0.00365445	Hemoglobin, gamma G
ARAP3	0.51	6.32 × 10^−7^	0.00377387	ArfGAP with RhoGAP domain, ankyrin repeat and PH domain 3
RBM43	−0.58	6.54 × 10^−7^	0.00377387	RNA-binding motif protein 43
ALAS2	0.72	9.99 × 10^−7^	0.00461308	Aminolevulinate, delta-, synthase 2
MOSC1	0.58	4.61 × 10^−6^	0.01776651	Mitochondrial amidoxime-reducing component 1
KLHDC8B	0.68	1.08 × 10^−5^	0.03566755	Kelch domain containing 8B
SNORD89	−0.31	1.73 × 10^−5^	0.04981533	Small nucleolar RNA, C/D box 89
PELI3	0.80	2.14 × 10^−5^	0.05501794	Pellino E3 ubiquitin protein ligase family member 3
TMEM150C	2.09	3.84 × 10^−5^	0.08875397	Transmembrane protein 150C
C10orf25	1.12	4.55 × 10^−5^	0.09544878	Chromosome 10 open reading frame 25

*FDR, false discovery rate*.

**Figure 1:**
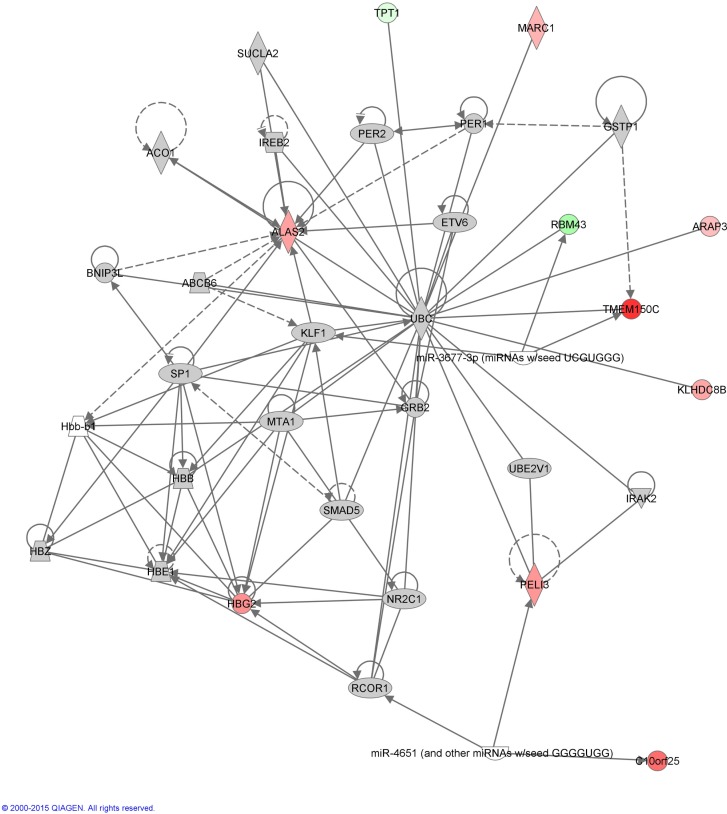
**Annotation enrichment analysis**. A network including the gene functions “cardiovascular system development and function,” “cell morphology,” and “hematological system development and function” was significantly enriched in patients with epilepsy after initiation of treatment with valproic acid [score = –log(*p*-value) = 30]. Red nodes designate up-regulated genes. Numbers indicate the log_2_ fold change (0 is equal expression). Uncolored nodes are genes in this network that were not in our list of differentially expressed genes.

Functional annotation of expression profiles was subsequently applied to identify functional changes in the context of genetic networks. A dataset containing 11 genes that had significant differential expression between before and after initiation of VPA treatment was uploaded into the Ingenuity Pathway Analysis software program. Only one genetic network was identified (enrichment score = 30), which included genes related to cardiovascular system development and function, cell morphology, as well as hematological system development and function (Table 3; Figure 1).

**Table 3:** **Network of genes significantly changed after treatment with valproic acid**.

Molecules in network	Score	Focus molecules	Top functions
ABCB6, ACO1, ALAS2, ARAP3, BNIP3L, C10orf25, ETV6, GRB2, GSTP1, HBB, Hbb-b1, HBE1, HBG2, HBZ, IRAK2, IREB2, KLF1, KLHDC8B, MARC1, miR-3677-3p (miRNAs w/seed UCGUGGG), miR-4651 (and other miRNAs w/seed GGGGUGG), MTA1, NR2C1, PELI3, PER1, PER2, RBM43, RCOR1, SMAD5, SP1, SUCLA2, TMEM150C, TPT1, UBC, UBE2V1	30	10	Cardiovascular system development and function, cell morphology, hematological system development and function

## Discussion

In this study, we examined VPA-induced changes in gene expression in the peripheral blood of patients with newly diagnosed epilepsy who were treated with therapeutic doses of the drug.

More than a decade ago, VPA was identified as a potent promotor of histone acetylation. Histone acetylation leads to relaxation of the nucleosome structure and transcriptional activation (17). In theory, HDAC inhibition could directly or indirectly affect between 2 and 5% of all genes (18). In mouse embryonic stem cells exposed to VPA, the expression levels of 2.4% of genes were altered by more than 1.5-fold (19). This property of VPA is believed to have an impact on embryogenesis (20), causing increased rate of major congenital malformations among children exposed to this drug *in utero* (21). Only one study has compared the genomic expression patterns in peripheral blood between epilepsy patients treated with VPA or without any drug. Expression levels of 461 genes were altered in VPA-treated compared to drug-free epilepsy patients; however, no information was provided regarding the VPA doses that were used (22).

Although we did not measure VPA-induced HDAC inhibition, the finding that a surprisingly small number of genes were differently expressed before and after the start of VPA treatment suggests that HDAC inhibition in our patients was very low or even absent. *In vitro*, HDAC inhibition is achieved at concentrations of 0.3–1.0 mM, corresponding to the serum drug concentration in patients treated with a daily dose of 20–30 mg/kg (17). According to the guidelines of epilepsy treatment in adults (23), we began VPA treatment in our patients at a minimal effective dose, with the mean daily dose of 11.4 ± 2.8 mg/kg. The lack of the HDAC inhibition in our study is probably related to the relatively low daily VPA dose that was used. Indeed, the risk of major congenital malformations in patients treated with VPA increases with doses above 700 mg/d and is pronounced with doses above 1500 mg/d (21), which are clearly higher than the doses used in our study (600–1200 mg/d). On the other hand, higher VPA doses than those used here were applied for the treatment of tumors (20 mg/kg; 1500 mg/d) to modulate gene expression via HDAC inhibition (24, 25). Therefore, to avoid the side effects of HDAC inhibition, it is recommended that patients be treated with the lowest effective dose of VPA.

During the evaluation period of about 3 months, no patient reported any epileptic seizures. However, the small sample size and short evaluation period prohibit us from making conclusions about whether seizure freedom was related to the VPA treatment or to the natural course of disease. Most patients had a presumed genetic etiology for their epilepsy, which usually suggests a self-limiting course. The anticonvulsive effect of VPA is mediated mainly through the reduced degradation, increased synthesis, decreased turnover, and, therefore, increased activity of gamma amino butyrate, and is not related to the alteration of gene expression (4). We could speculate that the anticonvulsive effect of VPA in our study was achieved without significant general activation of transcriptional activity.

After exposure to VPA, we observed changes in the expression levels of several genes (TPT1, ARAP3, and KLHDC8B) that are reported to be important in cancer-related pathways (26–28). Furthermore, the expression levels of two genes related to mitochondrial function, MOSC1 and ALAS2, were significantly changed. The product of MOSC1 localizes to the outer mitochondrial membrane and contributes to the regulation of nitric oxide synthesis (29). ALAS2 is an erythroid-specific, mitochondrially located enzyme that participates in heme biosynthetic pathways (30). Among the antiepileptic drugs, VPA has the highest potential to induce mitochondrial toxicity and could be even fatal in patients with some mitochondrial diseases. Use of VPA could be associated with the inhibition of respiratory chain complexes, impaired structural organization, and altered potential of the inner mitochondrial membrane (31, 32). Further investigations are needed to determine whether the differentially expressed genes identified in our study play roles in VPA-related mitochondrial toxicity.

Our study has several limitations. We had a relatively small sample of patients. However, the paired design of the study allowed us to minimize the influence of external factors and to find significant changes with this sample size. Our study does not account for the possible occurrence of other events that could influence gene expression during the evaluation period. The process of epileptogenesis starts long before the first seizure and continues during the disease course. This process could affect gene expression in the peripheral blood. The relatively low VPA dose received by study participants leaves open the possibility that the expressions of many genes were not influenced. On the other hand, the results probably reflect the gene expression profile in real clinical practice, when relatively low doses of VPA are used. Future studies should explore whether the use of higher VPA blood concentrations (e.g., VPA doses >1500 mg/d) significantly changes the gene expression profile. In this case, due to ethical issues, a different study design should be used, probably comparing gene expression in independent samples, such as patients treated with high VPA doses compared to patients treated with other antiepileptic drugs or healthy subjects. Another important limitation of our study was the absence of relevant clinical problems in our patients, besides epilepsy. It was not possible for us to correlate changes in gene expression with the potential side effects of the drug.

In conclusion, the findings of this study suggest that, at standard dosages, VPA modulates the expression of a surprisingly small number of genes. Further studies should be done to evaluate the effect of higher VPA dosages on gene expression. On the other hand, to avoid potential side effects of HDAC inhibition, we recommend that patients with epilepsy who are receiving this drug be treated with the lowest effective dose of VPA.

## Conflict of Interest Statement

The authors declare that the research was conducted in the absence of any commercial or financial relationships that could be construed as a potential conflict of interest.

## Abbreviations

HDAC, histone deacetylase; VPA, valproic acid.

## References

[1] Oun A Haldre S Magi M. Use of antiepileptic drugs in Estonia: an epidemiologic study of adult epilepsy. Eur J Neurol (2006) 13(5):465–70. 10.1111/j.1468-1331.2006.01268.x 16722970

[2] Belcastro V D’Egidio C Striano P Verrotti A. Metabolic and endocrine effects of valproic acid chronic treatment. Epilepsy Res (2013) 107(1–2):1–8. 10.1016/j.eplepsyres.2013.08.016 24076030

[3] Panayiotopoulos CP Atlas of Epilepsies. London, NY: Springer (2010).

[4] Chateauvieux S Morceau F Dicato M Diederich M. Molecular and therapeutic potential and toxicity of valproic acid. J Biomed Biotechnol (2010) 2010:479364. 10.1155/2010/479364 20798865 PMC2926634

[5] Shakespear MR Halili MA Irvine KM Fairlie DP Sweet MJ. Histone deacetylases as regulators of inflammation and immunity. Trends Immunol (2011) 32(7):335–43. 10.1016/j.it.2011.04.001 21570914

[6] Ornoy A. Valproic acid in pregnancy: how much are we endangering the embryo and fetus? Reprod Toxicol (2009) 28(1):1–10. 10.1016/j.reprotox.2009.02.014 19490988

[7] Chen Y Tsai YH Tseng SH. Valproic acid affected the survival and invasiveness of human glioma cells through diverse mechanisms. J Neurooncol (2012) 109(1):23–33. 10.1007/s11060-012-0871-y 22528797

[8] Verrotti A la Torre R Trotta D Mohn A Chiarelli F. Valproate-induced insulin resistance and obesity in children. Horm Res (2009) 71(3):125–31. 10.1159/000197868 19188736

[9] Qiao L Schaack J Shao J. Suppression of adiponectin gene expression by histone deacetylase inhibitor valproic acid. Endocrinology (2006) 147(2):865–74. 10.1210/en.2005-1030 16282359

[10] Eizirik DL Cardozo AK Cnop M The role for endoplasmic reticulum stress in diabetes mellitus. Endocr Rev (2008) 29(1):42–61. 10.1210/er.2007-0015 18048764

[11] Kakiuchi C Ishigaki S Oslowski CM Fonseca SG Kato T Urano F. Valproate, a mood stabilizer, induces WFS1 expression and modulates its interaction with ER stress protein GRP94. PLoS One (2009) 4(1):e4134. 10.1371/journal.pone.0004134 19125190 PMC2607540

[12] Chen B Wang JF Young LT. Chronic valproate treatment increases expression of endoplasmic reticulum stress proteins in the rat cerebral cortex and hippocampus. Biol Psychiatry (2000) 48(7):658–64. 10.1016/S0006-3223(00)00878-7 11032977

[13] Berg AT Berkovic SF Brodie MJ Buchhalter J Cross JH van Emde Boas W Revised terminology and concepts for organization of seizures and epilepsies: report of the ILAE Commission on Classification and Terminology, 2005-2009. Epilepsia (2010) 51(4):676–85. 10.1111/j.1528-1167.2010.02522.x 20196795

[14] McCarthy DJ Chen Y Smyth GK. Differential expression analysis of multifactor RNA-Seq experiments with respect to biological variation. Nucleic Acids Res (2012) 40(10):4288–97. 10.1093/nar/gks042 22287627 PMC3378882

[15] Robinson MD McCarthy DJ Smyth GK. edgeR: a Bioconductor package for differential expression analysis of digital gene expression data. Bioinformatics (2010) 26(1):139–40. 10.1093/bioinformatics/btp616 19910308 PMC2796818

[16] Storey JD Tibshirani R. Statistical significance for genomewide studies. Proc Natl Acad Sci U S A (2003) 100(16):9440–5. 10.1073/pnas.1530509100 12883005 PMC170937

[17] Gottlicher M Minucci S Zhu P Kramer OH Schimpf A Giavara S Valproic acid defines a novel class of HDAC inhibitors inducing differentiation of transformed cells. EMBO J (2001) 20(24):6969–78. 10.1093/emboj/20.24.6969 11742974 PMC125788

[18] Van Lint C Emiliani S Verdin E. The expression of a small fraction of cellular genes is changed in response to histone hyperacetylation. Gene Expr (1996) 5(4–5):245–53. 8723390 PMC6138027

[19] Boudadi E Stower H Halsall JA Rutledge CE Leeb M Wutz A The histone deacetylase inhibitor sodium valproate causes limited transcriptional change in mouse embryonic stem cells but selectively overrides polycomb-mediated Hoxb silencing. Epigenet Chromatin (2013) 6(1):11. 10.1186/1756-8935-6-11 23634885 PMC3769143

[20] Phiel CJ Zhang F Huang EY Guenther MG Lazar MA Klein PS. Histone deacetylase is a direct target of valproic acid, a potent anticonvulsant, mood stabilizer, and teratogen. J Biol Chem (2001) 276(39):36734–41. 10.1074/jbc.M101287200 11473107

[21] Tomson T Battino D. Teratogenic effects of antiepileptic drugs. Lancet Neurol (2012) 11(9):803–13. 10.1016/S1474-4422(12)70103-5 22805351

[22] Tang Y Glauser TA Gilbert DL Hershey AD Privitera MD Ficker DM Valproic acid blood genomic expression patterns in children with epilepsy – a pilot study. Acta Neurol Scand (2004) 109(3):159–68. 10.1046/j.1600-0404.2003.00253.x 14763951

[23] Schmidt D Schachter SC. Drug treatment of epilepsy in adults. BMJ (2014) 348:g254. 10.1136/bmj.g254 24583319

[24] Bilen MA Fu S Falchook GS Ng CS Wheler JJ Abdelrahim M Phase I trial of valproic acid and lenalidomide in patients with advanced cancer. Cancer Chemother Pharmacol (2015) 75(4):869–74. 10.1007/s00280-015-2695-x 25666183

[25] Avallone A Piccirillo MC Delrio P Pecori B Di Gennaro E Aloj L Phase 1/2 study of valproic acid and short-course radiotherapy plus capecitabine as preoperative treatment in low-moderate risk rectal cancer-V-shoRT-R3 (valproic acid – short radiotherapy – rectum 3rd trial). BMC Cancer (2014) 14:875. 10.1186/1471-2407-14-875 25421252 PMC4289397

[26] Kobayashi D Hirayama M Komohara Y Mizuguchi S Wilson Morifuji M Ihn H Translationally controlled tumor protein is a novel biological target for neurofibromatosis type 1-associated tumors. J Biol Chem (2014) 289(38):26314–26. 10.1074/jbc.M114.568253 25092287 PMC4176225

[27] Yagi R Tanaka M Sasaki K Kamata R Nakanishi Y Kanai Y ARAP3 inhibits peritoneal dissemination of scirrhous gastric carcinoma cells by regulating cell adhesion and invasion. Oncogene (2011) 30(12):1413–21. 10.1038/onc.2010.522 21076469

[28] Krem MM Luo P Ing BI Horwitz MS. The kelch protein KLHDC8B guards against mitotic errors, centrosomal amplification, and chromosomal instability. J Biol Chem (2012) 287(46):39083–93. 10.1074/jbc.M112.390088 22988245 PMC3493949

[29] Klein JM Busch JD Potting C Baker MJ Langer T Schwarz G. The mitochondrial amidoxime-reducing component (mARC1) is a novel signal-anchored protein of the outer mitochondrial membrane. J Biol Chem (2012) 287(51):42795–803. 10.1074/jbc.M112.419424 23086957 PMC3525010

[30] Fujiwara T Harigae H. Pathophysiology and genetic mutations in congenital sideroblastic anemia. Pediatr Int (2013) 55(6):675–9. 10.1111/ped.12217 24003969

[31] Nanau RM Neuman MG. Adverse drug reactions induced by valproic acid. Clin Biochem (2013) 46(15):1323–38. 10.1016/j.clinbiochem.2013.06.012 23792104

[32] Pourahmad J Eskandari MR Kaghazi A Shaki F Shahraki J Fard JK. A new approach on valproic acid induced hepatotoxicity: involvement of lysosomal membrane leakiness and cellular proteolysis. Toxicol In Vitro (2012) 26:545–51. 10.1016/j.tiv.2012.01.020 22342442

